# A twenty year journey to understand how ATP activates guanylyl cyclase A and B

**DOI:** 10.1186/2050-6511-14-S1-O13

**Published:** 2013-08-29

**Authors:** Lincoln R Potter

**Affiliations:** 1 Department of Biochemistry Molecular Biology and Biophysics University of Minnesota Minneapolis MN USA

## Background

ATP was first shown to enhance the activity of natriuretic peptide (NP)-stimulated guanylyl cyclase (GC)-A in 1987 [1]. Later, NP-stimulation of GC-A and GC-B was reported to be dependent on ATP and a model was proposed where ATP binding to the kinase homology domain elevates maximal velocity of these enzymes [2]. Beginning in 1992, we demonstrated that GC-A and GC-B are phosphorylated and that phosphorylation is required for NP-stimulation [3-6]. Subsequently, ATP was shown to increase the phosphorylation and activity of GC-A in vitro, which explained why ATP is required for their activation [7]. Later, we demonstrated that ATP is not required for NP-dependent activation of GC-A and GC-B if phosphatase inhibitors are included in the assay and substrate levels are high [8]. Surprisingly, we found that ATP dramatically reduced the Michaelis constants (Kms) for GC-A and GC-B but had no effect on their maximal velocities [9].

## Results

Recent studies have determined how ATP allosterically activates GC-A and GC-B [10]. In the absence of ATP, NPs activated these enzymes > 10-fold in a positive cooperative manner. In the absence of NP, ATP shifted the substrate-velocity profiles from cooperative to linear but did not change the Km. In the presence of NPs, ATP competed with GTP for binding to an allosteric site, which enhanced the activation of GCs by decreasing the Km. Thus, NP binding was required for communication of the allosteric activation signal to the catalytic site. Concentration-response assays determined that the ability of ATP to activate GCs decreased and enzyme potency increased with increasing GTP concentrations, consistent with reciprocal regulation of the allosteric and catalytic sites. Point mutations in the purine-binding site of the catalytic domain abolished GC activity but not allosteric activation. Co-expression of inactive mutants produced half the activity expected for symmetric catalytic dimers. 2’deoxy-ATP and 2’deoxy-GTP were poor allosteric activators, but 2’-deoxy-GTP was an effective substrate, consistent with distinct binding requirements for the allosteric and catalytic sites.

## Conclusion

GC-A and GC-B are asymmetric homodimers with distinct and reciprocally regulated catalytic and allosteric sites that bind GTP and ATP, respectively.
